# MADet: A Multi-Dimensional Feature Fusion Model for Detecting Typical Defects in Weld Radiographs

**DOI:** 10.3390/ma18153646

**Published:** 2025-08-03

**Authors:** Shuai Xue, Wei Xu, Zhu Xiong, Jing Zhang, Yanyan Liang

**Affiliations:** 1 School of Computer Science and Engineering, Faculty of Innovation Engineering, Macau University of Science and Technology, Macau 999078, China; 2 School of Applied Science and Civil Engineering, Beijing Institute of Technology, Zhuhai 519000, China; 3 The Institute for Sustainabele Development, Macau University of Science and Technology, Macau 999078, China; 4 Office of Academic Affairs, Beijing Institute of Technology, Zhuhai 519000, China; 5 Aerospace and Informatics Domain, Beijing Institute of Technology, Zhuhai 519000, China

**Keywords:** weld defect detection, X-ray image analysis, multi-scale feature fusion, attention mechanism, deep learning

## Abstract

Accurate weld defect detection is critical for ensuring structural safety and evaluating welding quality in industrial applications. Manual inspection methods have inherent limitations, including inefficiency and inadequate sensitivity to subtle defects. Existing detection models, primarily designed for natural images, struggle to adapt to the characteristic challenges of weld X-ray images, such as high noise, low contrast, and inter-defect similarity, particularly leading to missed detections and false positives for small defects. To address these challenges, a multi-dimensional feature fusion model (MADet), which is a multi-branch deep fusion network for weld defect detection, was proposed. The framework incorporates two key innovations: (1) A multi-scale feature fusion network integrated with lightweight attention residual modules to enhance the perception of fine-grained defect features by leveraging low-level texture information. (2) An anchor-based feature-selective detection head was used to improve the discrimination and localization accuracy for five typical defect categories. Extensive experiments on both public and proprietary weld defect datasets demonstrated that MADet achieved significant improvements over the state-of-the-art YOLO variants. Specifically, it surpassed the suboptimal model by 7.41% in mAP@0.5, indicating strong industrial applicability.

## 1. Introduction

Welding is a core process in key areas such as aerospace, pressure vessel manufacturing and shipbuilding. The relevant assembled components need to operate under specific working conditions, and the quality of the welds is strictly required [1]. The structural performance of the final component directly depends on the quality of the welds–internal defects may cause stress concentration, resulting in mechanical performance degradation and shortened service life [2,3,4]. Therefore, it is crucial to implement effective inspection to reduce the risk of component failure. Manual visual inspection is easily affected by subjective factors [5,6], and destructive inspection is only applicable to sampling analysis. Therefore, non-destructive testing (NDT) technology is widely used to achieve non-destructive evaluation of the internal structure of the weld while ensuring the integrity of the component [7,8,9].

Among the many non-destructive testing methods [10,11], X-ray flaw detection (RT) is widely used because it can generate two-dimensional images of the internal structure of the weld [12,13,14]. This technology records the difference in the absorption of radiation by the material, so that volume defects (such as pores, slag inclusions) and cracks are presented on the X-ray film in the form of grayscale changes [15,16]. The early methods for realizing automatic analysis of radiographic images mainly rely on traditional computer vision technology [17], usually manual feature extraction is performed first, and then models such as support vector machines (SVM) [18] or artificial neural networks (ANN) [19] are used for classification. However, faced with the complexity of industrial scenarios and the diversity of defects, such methods often have poor generalization capabilities.

With the emergence of deep learning, especially Convolutional Neural Networks (CNN) [20], a new solution to this problem has been found. This type of end-to-end model can automatically learn feature representations from raw images. Although it has achieved remarkable results in many applications, it still faces several challenges in the field of weld radiographic inspection: First, the CNN model has limitations in the detection of small-sized and low-contrast defects. Although continuous downsampling operations can capture high-level semantic information, they will lose fine-grained spatial information that helps detect tiny features; second, it is challenging to learn accurate classification and positioning from complex weld area background textures and defect morphology diversity. In addition, the technical trade-off between speed and accuracy also hinders practical applications–although the two-stage detector (such as Faster R-CNN) [21] has high accuracy, its computational workload is difficult to meet the online detection requirements of high-throughput scenarios; and although the single-stage detector of the YOLO series has fast inference speed, the standard architecture has insufficient detection accuracy for small-sized and low-contrast weld defects. This study is committed to designing a detection model that can simultaneously meet the requirements of industrial quality inspection inference speed and detection accuracy.

To address the above challenges, this paper proposes a weld defect detection model based on X-ray images, the multi-dimensional feature fusion model (MADet). The model is built on the YOLO framework, which improves the detection capability of complex defects (such as small low-contrast defects) while maintaining a lightweight structure that adapts to high-speed reasoning. The main innovative contributions include:

- A new weld defect detection model, the multi-dimensional feature fusion model (MADet), is proposed. The model integrates grouped convolution, hybrid attention mechanism and residual structure to enhance the feature extraction capability of small-sized and edge-blurred defects under complex backgrounds.
- A multi-dimensional feature fusion module (MDFFM) is designed to hierarchically integrate cross-scale semantic information. This module is designed to enhance feature representation at different resolutions and improve the robustness of the model in identifying various defects.
- An anchor-based detection head is adopted. This component aims to achieve collaborative modeling from local details to global semantics to enhance feature focusing, target positioning and recognition capabilities.

## 2. Materials and Methods

### 2.1. Acquisition and Preparation of Radiographic Images of Butt Welds

**Figure 1:**
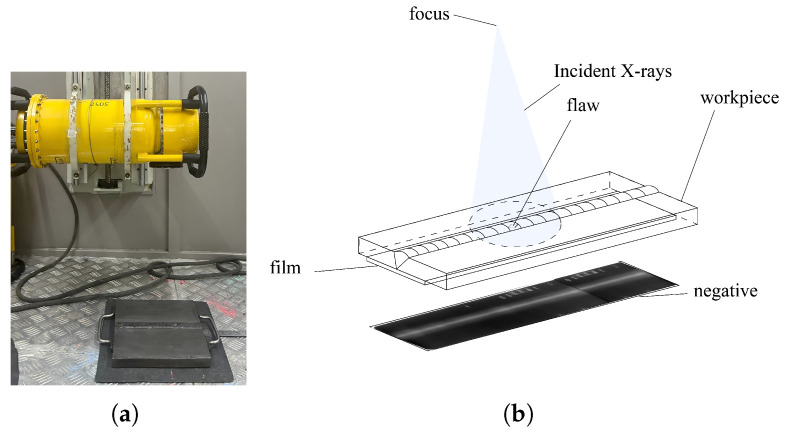
This is a wide figure. The schemes followed the same format. If there are multiple panels, they should be listed as: (**a**) Field inspection images of X-ray welding seams. (**b**) X-ray radiograph of weld seam.

X-ray radiography was performed on steel plate butt welds using a film-based imaging technique. During the inspection process, industrial X-ray films were placed on one side of the welded joint, whereas the X-ray source was positioned on the opposite side, which enabled through-transmission imaging. After exposure, the films were developed using standard chemical processing techniques to reveal the internal features of the welds. We employed a portable X-ray flaw detector (Model XXG-2505, manufactured by Tongda Technology Co., Ltd., Dandong, Liaoning, China) for radiographic testing. The X-ray system has a penetration capability of 0–50 mm in steel, as shown in Figure 1.

To facilitate digital processing and analysis, radiographic films were digitized using a high-resolution X-ray film scanner. The resulting electronic images preserved the grayscale and structural details required for the defect detection and classification.

In addition to the experimentally acquired images, supplementary radiographic images were collected from publicly available online sources to enhance the dataset diversity. All images, both scanned and collected, were carefully annotated by level-2 NDT experts, who labeled common weld defects such as porosity, slag inclusion, lack of fusion, and cracks. This annotated dataset was used to train and evaluate the weld defect detection algorithms.

### 2.2. Weld Defect Types and Characteristics

**Figure 2:**
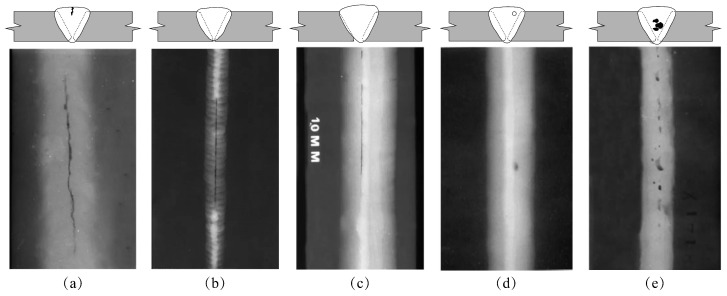
The X-ray radiographs of the weld seams: (**a**) Cracks. (**b**) Slag Inclusions. (**c**) Incomplete Penetration. (**d**) Porosity. (**e**) Lack of Fusion.

This study focuses on five typical internal defects prevalent in industrial welding: Cracks, Incomplete Penetration, Incomplete Fusion or Lack of Fusion (LOF), Porosity, and Slag Inclusions. X-ray radiographs of the weld seams are shown in Figure 2. Understanding their formation mechanisms and radiographic signatures is critical for subsequent dataset annotation, model training, and validation. Table 1 summarizes the main causes of the five typical weld defects mentioned above and their characteristics in X-ray images.

### 2.3. MADet Overview

**Figure 3:**
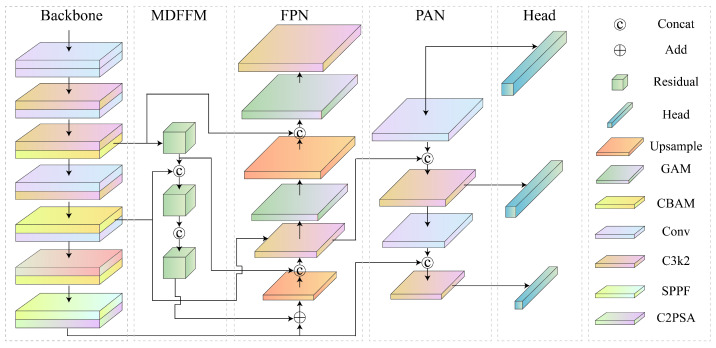
MADet Network Structure Diagram.

As illustrated in Figure 3, an object detection model, MADet, specifically designed to identify typical defects in weld radiographic images, was proposed. MADet is based on the end-to-end detection architecture of YOLOv11 and establishes an efficient multiscale feature extraction and fusion mechanism to enhance the detection accuracy and robustness of defect targets of varying scales.

MADet retains the lightweight backbone network structure of YOLOv11 and integrates a hybrid attention mechanism and feature-sharing strategies to augment its capability of modeling multi-scale features and small object characteristics. Specifically, the model concurrently transmits shallow-layer texture features to the Feature Pyramid Network (FPN), Path Aggregation Network (PAN), and Multi-Dimension Feature Fusion Module (MDFFM), thereby achieving efficient sharing of low-level detailed information. The FPN and PAN employ bottom-up and top-down feature fusion pathways, respectively, to amalgamate spatial information from different hierarchical levels, consequently improving the perceptual ability and detection accuracy of the model for small defect targets. The MDFFM focuses on lateral multi-scale semantic fusion to strengthen the discriminative power and contextual modeling capabilities of high-level features, and further optimizes the shallow-layer feature representation. Ultimately, MADet outputs three sets of feature maps with distinct spatial resolutions and complementary semantics. These maps constitute a unified multiscale detection branch corresponding to the detection tasks for small, medium, and large targets, respectively. This design aims to improve the detection performance and robustness of the model in complex weld defect detection.

### 2.4. Backbone and MDFFM

**Figure 4:**
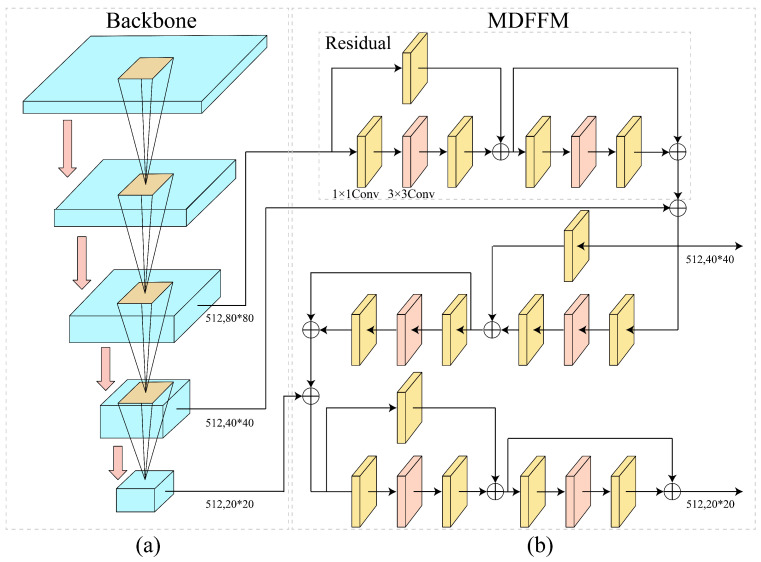
Core structure diagram of MADet. (**a**) Backbone of MADet (**b**) Multi-Dimensional Feature Fusion Module (MDFFM) of MADet.

As depicted in Figure 4, the core structure of the MADet comprises a backbone network and a Multi-Dimension Feature Fusion Module (MDFFM). The backbone network employs five layers of group convolutions to reduce computational complexity and redundant parameters, thereby enhancing computational efficiency while preserving feature extraction capabilities. Following each group convolution layer, a C3K2 module based on residual connections and cross-channel convolutions was incorporated. This module combines the original features with channel interactions to enhance the extraction of texture and edge information, thereby improving the quality of shallow-layer feature representation. Under the feature-sharing strategy, three sets of feature maps with different resolutions output by the backbone network were fed into the MDFFM module. Two sets of discriminative multi-scale semantic features were generated through lateral connections and local-global channel interaction operations. Finally, these enhanced semantic features, along with the original multi-scale features, were jointly input into the FPN. This process aims to improve the precise modeling and semantic representation of typical defect regions in weld radiographic images, providing stable and rich features for multi-scale defect detection in weld radiographic images.

### 2.5. Backbone and MDFFM

During the model training phase, data-augmented images were fed in batches (Batch Size) into the backbone network of the MADet. Initially, features pass sequentially through two layers of group convolutions with a 3 × 3 kernel to achieve channel compression and dimensional adjustment, and subsequently enter the first C3K2 module to construct preliminary global semantic information. Subsequently, the features traverse another set of group convolutions and a C3K2 module to further enhance contextual awareness, with cross-channel interaction and residual fusion mechanisms introduced. After processing using the third group convolution and C3K2 module, a Convolutional Block Attention Module [22] (CBAM) is incorporated to enable joint attention regulation across channel and spatial dimensions, outputting the first shared feature map (512 × 80 × 80). This feature map continues through another group convolution and C3K2 module, with the CBAM again introduced to generate the second feature map (512 × 40 × 40). In a similar manner, the third feature map is obtained via further group convolutions and C3K2 modules, followed by multi-scale context fusion using Spatial Pyramid Pooling-Fast) and C2PSA (Cross-Scale Channel Attention) modules. Ultimately, three sets of feature maps with varying resolutions and deep semantic complementarity were produced, providing unified and high-quality multi-scale semantic support for the subsequent MDFFM and FPN networks, facilitating the precise detection of multi-scale defect target.

As illustrated in Figure 4b, the MDFFM is primarily composed of identity and convolution blocks, which mainly serve to fuse features from different hierarchical levels and construct cross-scale semantic feature maps with image discriminability, thereby enhancing detection accuracy and model robustness. Initially, a large-scale feature map output by the backbone network was input into the residual structure. Channel compression is achieved via an Identity Block, preserving critical texture information while minimizing information loss and reducing computational overhead. Subsequently, the dimensionally reduced feature map enters multiple stacked Convolution Blocks. Through standard convolutions and nonlinear activation functions, the local context and residual information are fused to establish lateral connections between multi-scale semantics. This enhances the information flow and complementarity among features of different resolutions, compensating for deficiencies in the shallow-layer semantic expression. The fused features were then combined with the medium-scale features from the second layer of the backbone network, achieving cross-level information fusion while retaining the spatial structural textures and semantic abstract features. Subsequently, the fused result is combined with the deep-layer features from the third layer of the backbone network to further strengthen the high-order semantic expression capability. Finally, the MDFFM outputs two sets of discriminative semantic feature maps, providing a feature reference for the precise localization and classification of weld defects.

### 2.6. Hybrid Attention Mechanism

To enhance the model’s capability to localize and identify weld defects, a hybrid attention mechanism incorporating the CBAM and GAM [23] was introduced into the backbone network and FPN of the MADet, respectively. By fusing channel and spatial attention, this mechanism guides the model to focus on more discriminative semantic features, thereby improving the detection accuracy and robustness.

**Figure 5:**
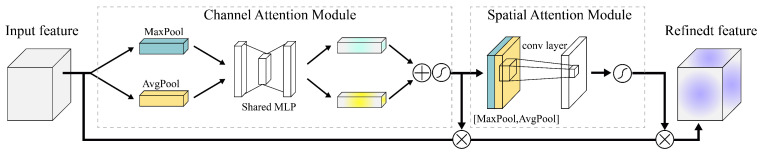
CBAM Hybrid Attention Module.

The backbone network is primarily responsible for extracting the shallow layer features. Although it captures rich texture details and edge information, it often lacks effective representation of deep semantic structures, particularly when dealing with challenges in weld radiographic images, such as low contrast, small scale and blurred edges. Therefore, the CBAM, as shown in Figure 5, the hybrid attention module was integrated into the output feature maps of each level of the backbone network. Through its channel attention mechanism, the CBAM screens the texture features with significant discriminative power. Its spatial attention mechanism further enhances the sensitivity to faint defect regions, thereby refining the perceptual ability of the model for shallow-layer features. Furthermore, to overcome the propensity of the FPN to generate redundant or conflicting information during cross-scale fusion, a GAM global attention mechanism is embedded after each fusion stage. By uniformly modeling across the channel and spatial dimensions, the GAM dynamically optimizes the weight distribution of the fused features, effectively strengthening the semantic consistency and contextual awareness. This improves the robustness of the model under complex background conditions and its ability to accurately identify defects of different scales.

CBAM is primarily composed of a channel attention module and a spatial attention module connected in series.During feature propagation, the feature map first passes through the channel attention module. Global max pooling and global average pooling operations were performed on the feature map to generate two distinct global descriptor vector sets. These vectors were processed separately using a shared multi-layer perceptron (MLP) and then added together to produce a channel-wise attention weight vector using a sigmoid function. This vector is then multiplied channel-wise with the original feature map to select and emphasize important channel features with discriminative power. Subsequently, the channel-attention-weighted feature map is passed to the spatial attention module. This module first performs max and average pooling along the channel dimension, concatenates them along the spatial dimension, and then fuses them through a convolution operation to generate a spatial attention map. This map, also processed by a sigmoid function, is multiplied element-wise with the input features, ultimately significantly enhancing the sensitivity to potential defect regions in the spatial domain, thereby generating a more refined attention-enhanced feature representation of the input image.

**Figure 6:**
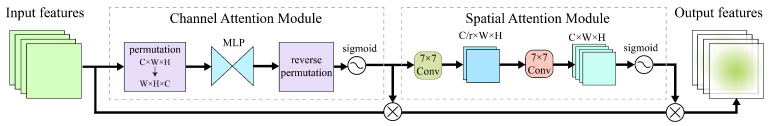
GAM Hybrid Attention Module.

The feature propagation process of the GAM is shown in Figure 6, which emphasizes the unified modeling of the channel and spatial dimensions. First, the input feature map underwent global average pooling in the spatial dimension to generate channel-level global descriptors for the feature map. Two serially connected fully connected (FC) layers are then used to model the inter-channel dependencies and capture channel-level semantic information. Second, a convolution operation related to the spatial position achieves local modeling in the spatial dimension, capturing the contextual correlations of spatial locations within the corresponding feature map. Subsequently, the fusion of the channel and spatial information through a sigmoid function yields comprehensive attention weights. These weights dynamically adjust the response intensity of each channel and spatial position in the feature map, ultimately forming a uniformly adjusted feature map. This effectively reduces redundant information and conflicts during feature fusion, enhances semantic consistency and contextual awareness, and provides precise and robust feature representations for subsequent multi-scale object detection.

### 2.7. Head

To further enhance the localization accuracy of MADet and its ability to recognize small targets in weld defect detection tasks, an anchor-based detection head was employed, replacing the anchor-free structure originally present in the YOLOv11 architecture. Although anchor-free methods offer the advantages of directly classifying and regressing target center points, possessing a relatively simple overall structure, and having fewer parameters, they lack the spatial guidance information provided by anchor box priors. This deficiency makes it difficult for the model to effectively establish accurate spatial distributions of target regions in high-resolution and complex scenarios, particularly when processing typical defects in weld radiographic images characterized by low contrast, small size, and blurred edges, often leading to localization deviations or even missed detections.

To address these issues, an anchor-based detection head was introduced. By predefining multiple candidate anchor boxes with varying scales and aspect ratios on the feature map, the network is guided to explicitly learn the positional offset relationships between the targets and anchor boxes, thereby establishing spatial prior information for the targets. The anchor-based mechanism provides a set of explicit and stable candidate regions from the outset of model training, prompting the model to learn precise localization and feature capture methods for small targets. Concurrently, the multiscale anchor box design effectively covers diverse variations in the target scales within the image, which helps enhance the adaptability of the model to defects of different sizes and morphologies.

Furthermore, the introduction of the anchor mechanism provides a clear criterion for distinguishing positive and negative samples, effectively reducing ambiguity in target region definition and significantly improving the model’s target recall capability in complex backgrounds.In summary, by adopting an anchor-based multi-scale anchor box mechanism, MADet significantly enhances the recognition accuracy of small targets and overall detection robustness in complex weld defect scenarios. It overcomes the problems of unstable localization and high miss rates for small-scale defects associated with traditional anchor-free methods, ultimately achieving a more precise and robust object-detection performance.

### 2.8. Loss Function

To achieve accurate detection and classification of weld defect targets, MADet adopts a multitask joint optimization loss function that combines target box regression loss, target positioning loss, and category classification loss.

The target box regression loss is mainly used to measure and optimize the position deviation and size difference between the anchor box predicted by the network and the real annotation box. The traditional Intersection over Union (IoU) loss only focuses on the degree of overlap between the predicted and real boxes and fails to fully express the difference in spatial position and size ratio between the two boxes, which can easily lead to inaccurate target positioning, especially for small-scale or defective targets with significant shape differences. Therefore, MADet uses the CIoU loss function as the target box regression loss calculation function. CIoU not only takes into account the target overlapping area, but also pays attention to the distance between the center points and the difference in aspect ratio, thereby achieving more refined positioning capabilities, as shown in Equation (1).

(1) $$L_{\mathrm{box}}=1-\mathrm{IoU}+\frac{\rho^{2}\left(b,b^{bt}\right)}{c^{2}}+\alpha v,$$

where IoU denotes the intersection over union between the predicted box *b* and the ground truth box $b^{bt}$. ρ represents the Euclidean distance between the centers of the two boxes. *c* is the diagonal length of the smallest enclosing rectangle that covers both the boxes. α is a trade-off parameter for aspect ratio deviation. where *v* is the aspect ratio consistency metric.

The target confidence loss is used to measure whether there is a target in each predicted anchor box, and binary cross-entropy loss (BCE) is used, as shown in Equation (2).

(2) $$L_{obj}=-\sum_{i=1}^{S^{2}}\sum_{j=1}^{A}\left[p_{ij}^{gt}\log{\hat{p}}_{ij}+\left(1-p_{ij}^{gt}\right)\log\left(1-{\hat{p}}_{ij}\right)\right]$$

Among them, Where, $S^{2}$ represents the total number of grid cells in the feature map, *A* denotes the number of anchor boxes per cell, $p_{ij}^{gt}$ indicates the ground-truth label of the *j*-th anchor box in the *i*-th cell, and ${\hat{p}}_{ij}$ is the predicted probability of target presence.

The category classification loss is used to quantify the difference between the predicted target category probability and the true category label, and uses multi-category Cross Entropy Loss (cross entropy loss), as shown in Equation (3).

(3) $$L_{obj}=-\sum_{i=1}^{S^{2}}\sum_{j=1}^{A}\sum_{c=1}^{C}y_{ijc}^{gt}\log\left({\hat{y}}_{ijc}\right),$$

Among them, *C* represents the number of categories, $y_{ijc}^{gt}$ is the one-hot encoded label of the true category, and ${\hat{y}}_{ijc}$ is the category probability predicted by the network.

Finally, the overall loss function of MADet is the weighted sum of the target box regression loss, target positioning loss, and category classification loss, as shown in Equation (4).

(4) $$L_{total}=\lambda_{box}L_{box}+\lambda_{obj}L_{obj}+\lambda_{cls}L_{cls},$$

Among them, $\lambda_{box}$, $\lambda_{obj}$ and $\lambda_{cls}$ represent the weight coefficients of the box regression, target confidence, and category classification losses, respectively. To balance the optimization of the three objectives, the weight coefficients were set to 1 to optimize the model performance.

## 3. Experiment

### 3.1. Experimental Platform

All experiments were conducted using models implemented in the PyTorch 2.7.1 framework. Model training and testing were performed on a high-performance server running Ubuntu 20.04, equipped with an Intel Xeon Platinum 8375C CPU 2.90 GHz (128 threads) and eight NVIDIA RTX A6000 GPUs (48 GB each). The GPU driver version was 535.183.01, and the CUDA version was 12.2. The optimizer for loss in this study was Adamw, and the learning rate decreased in steps. The maximum and minimum learning rates for the initialization were 0.01 and 0.0001, respectively. The upper and lower limits of the learning rate were 0.001 and 0.0001, respectively. The model parameters are presented in Table 2.

### 3.2. Dataset and Preprocessing

**Figure 7:**
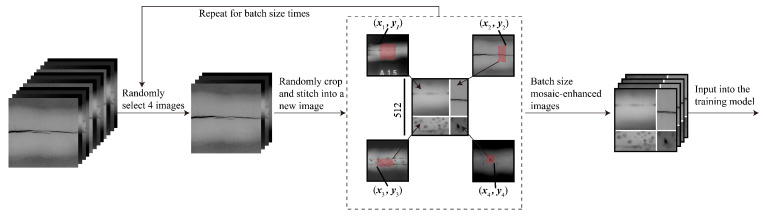
Mosaic enhancement schematic diagram.

The dataset distribution is shown in Table 3. A total of 6379 weld defect images in five categories were produced and collected, and the data were divided into three sets–training, validation, and test–in a ratio of 8:1:1 according to each category. To alleviate the problems of small target size, irregular shape, blurred edges, and complex background of sample weld defects, the mosaic enhancement method was introduced to build a richer and more complex training dataset, thereby enhancing the stability of the model and reducing the dependence of model training on data volume. Specifically, the mosaic enhancement method takes one image from the training set from the front to the back, randomly takes three images from the remaining dataset, randomly scales and crops them, divides them into four quadrants with a randomly generated intersection as the center, and splices them into a complete image in sequence. While the image content is synthesized, the corresponding bounding box label is also adjusted synchronously according to the scaling ratio and spatial offset to ensure the spatial accuracy of the target annotation, as shown in Figure 7.

### 3.3. Comparative Experiment

To verify the detection capability of MADet in the weld defect detection task, this study selected the mainstream YOLOv5 [24], YOLOv6 [25], YOLOv8, YOLOv9, YOLOv10 [26], YOLOv11 [27], and YOLOv12 [28] target detection models in Ultralytics as the comparison benchmarks, and evaluated the performance on five types of defects, namely CK, LOF, LOP, Por, and SI, and used mAP0.5 [29] as the overall detection performance measurement indicator. The detection results for each model on the test set are presented in Table 4.

As shown in the table, MADet has significant advantages for all types of defect targets. Among them, the detection accuracy of the five types of defects, CK, LOF, LOP, Por, and SI, reached 94.22%, 90.96%, 90.85%, 94.73%, and 94.49%, respectively, which is still significantly improved in multiple categories compared with the best baseline models, YOLOv9 and YOLOv11. For example, in the CK category, the detection accuracy of YOLOv9 was 81.91%, whereas the proposed method improved it to 94.22%. In the LOP category, YOLOv9 achieved 78.94%, whereas the proposed method improved to 90.85%. Simultaneously, in terms of the overall performance index mAP@0.5, the proposed method achieved 91.05%, which is significantly better than the current suboptimal YOLOv9 model of 81.42%, proving the effectiveness of the proposed method in terms of its accuracy and robustness. In summary, the model in this study not only achieves a breakthrough in the overall detection performance but also shows superior generalization and discrimination capabilities in the detection of multiple types of defective targets, particularly in the recognition of small targets and targets with blurred edges.

### 3.4. Ablation Experiment

To further verify the contribution of the proposed module to improving the performance of weld defect detection, this study designed a set of ablation experiments under the YOLOv11 framework, gradually introduced different improved modules by controlling the variable method, and evaluated their effects on five types of defects and the overall detection performance of the test set. The experimental results are listed in Table 5.

The baseline model was the original YOLOv11, whose detection head was the default, anchor-free architecture. The average detection accuracy of each category was low, and the mAP@0.5 was 81.42%. To improve the model’s ability to perceive the target location, the detection head was first replaced with an anchor-based structure, which can provide more robust positioning capabilities in actual industrial inspections. After the replacement, the model achieved significant gains in the four types of defects, Ck, LOP, Por, and SI, and the overall mAP increased to 87.07%, verifying the applicability of the anchor-based mechanism for this task.

To further enhance the model, this study introduces a Convolutional Block Attention Module (CBAM) to enhance the model’s ability to focus on salient areas, allowing the model to focus on more discriminative feature areas. In the CBAM-enhanced data, the model’s detection performance in categories such as LOF, Por, and SI was significantly improved, with the mAP increasing to 88.55%. On this basis, the global attention mechanism (GAM Global Attention Module) was further introduced to build joint attention, further improving the model’s context modeling capabilities, so that mAP was increased to 89.07%, and performance was further optimized in most categories.

Finally, the MADet model, which combines the anchor box detection head and dual attention mechanism, achieved the best performance in all defect categories, which was nearly 10% higher than the original YOLOv11, fully verifying the effectiveness of the proposed module in improving the accuracy and robustness of the target detection.

### 3.5. Performance Visualization

**Figure 8:**
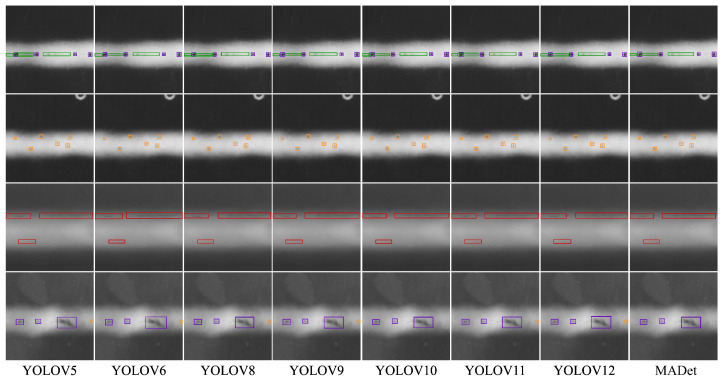
Visualization of multiple model detections.

**Figure 9:**
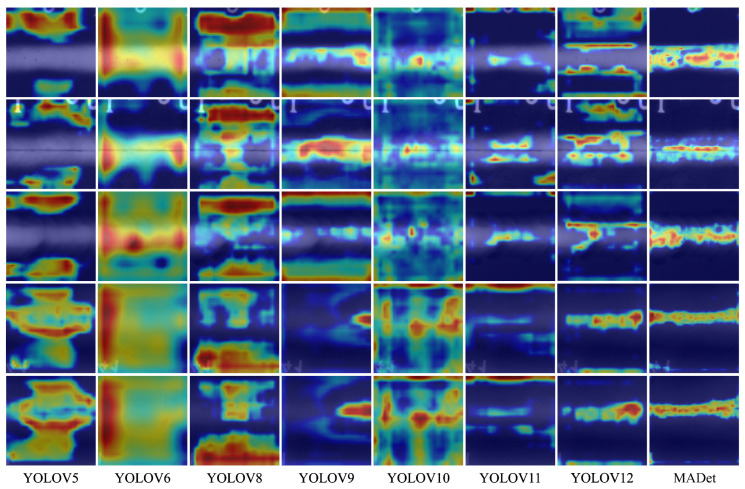
Response heat map of the target area.

To further compare the perception capabilities of different detection models for weld defect areas, this study visualized the intermediate features of each model using Grad-CAM and drew the response heat map of the target area, as shown in Figure 8 and Figure 9. It can be observed from the figure that the YOLOv5–YOLOv8 models generally have problems with response area offset and unclear diffusion, the model activation range is large, and the focus area often covers the non-defect area, indicating that they have insufficient perception capabilities for the boundaries and morphology of defects. In particular, although YOLOv6 and YOLOv8 have high responses to certain areas, obvious misactivations remain, resulting in unstable detection results. With the evolution of the model version from YOLOv9 to YOLOv12, the high-response areas in the heat map gradually concentrated on the defect location, indicating that the model had enhanced perception ability. However, there are still problems, such as local blur or incomplete response, especially for small-scale defects or edge-blurred targets, and the response range is easily disturbed by the background. In contrast, MADet can accurately focus on the main defect area for all defect samples. The high-response area in the heat map is highly consistent with the actual defect morphology, with clear boundaries and concentrated responses, which is significantly better than the other benchmark models. This demonstrates that the proposed multidimensional feature fusion strategy and attention mechanism can effectively enhance the model perception of key areas and improve the discrimination performance of small defect targets in complex backgrounds.

**Figure 10:**
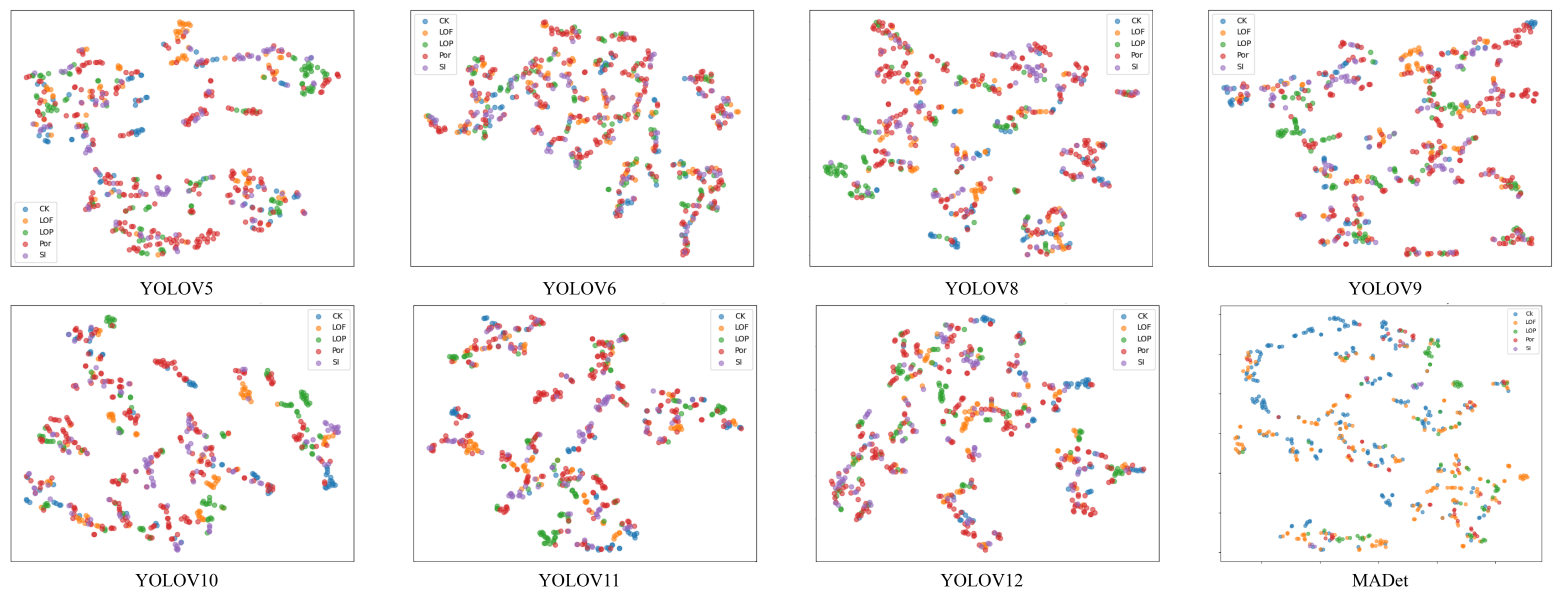
Comparison of Intermediate Feature Activation Maps of Multiple Models.

In addition, the high-dimensional features extracted by each model were reduced in dimension by the t-distributed Stochastic Neighbor Embedding (t-SNE) method to explore the discrimination ability of different models for weld defect categories and the separability of the feature embedding space, as shown in Figure 10. It can be observed from the figure that the samples of each category of early models, such as YOLOv5 and YOLOv6, are highly mixed in the low-dimensional space, and the boundaries between different categories are blurred, indicating that their feature expression ability is weak and that it is difficult to achieve an effective category distinction. Although the feature clustering effect of subsequent versions, such as YOLOv8–YOLOv10, has improved, there is still a certain degree of category overlap, especially in visually easy-to-mix categories, such as LOF and LOP, and SI and Por. The classification boundaries remain unclear, indicating that the model’s ability to distinguish complex defect categories is limited. With the optimization of the model structure, YOLOv11 and YOLOv12 showed a good trend of inter-class separation, and the feature points gradually showed category-centered clustering characteristics in space. However, drift still occurred in a small number of samples in some categories, indicating that the recognition of some edge or blurred targets remained uncertain. In contrast, the MADet model exhibited the best feature distribution performance. Samples of each category formed a highly separated and clearly structured cluster distribution in two-dimensional space, with obvious boundaries between classes and high compactness within classes, which fully demonstrated the strong generalization ability and robustness of the model in feature expression and category discrimination. In particular, for categories that are easily confused, such as LOF and LOP, clear and distinguishable feature clusters can be formed, verifying the effectiveness of the designed multi-dimensional feature fusion and attention mechanism in enhancing the feature discriminability.

## 4. Conclusions

This paper presented MADet, a novel framework for weld defect detection in X-ray radiographic images, designed to address persistent challenges such as small defects, low contrast, and high background noise. The main conclusions and contributions of this work are summarized as follows:

- A Novel Multi-Dimensional Fusion Architecture: A new model, MADet, was developed based on YOLOv11 architecture. It introduces several key enhancements, including a Multi-Dimensional Feature Fusion Module (MDFFM) to bridge the semantic gaps between features, a hybrid attention mechanism (CBAM and GAM) for better feature discrimination, and an anchor-based detection head to improve the localization accuracy for small or indistinct defects.
- State-of-the-Art Performance: Through extensive experiments on a custom dataset of five typical weld defect types, MADet demonstrated superior performance. It achieved a mean Average Precision (mAP@0.5) of 91.05%, outperforming the strongest baseline model (YOLOv9) by nearly 10%.
- Validated Component Efficacy: The effectiveness of each proposed architectural component was systematically confirmed through comprehensive ablation studies. Furthermore, qualitative analysis using Grad-CAM and t-SNE visualizations verified that MADet achieves more precise defect localization and stronger feature discrimination compared to existing state-of-the-art models.
- Strong Industrial Applicability: The combination of high accuracy, particularly for challenging defect types, and a robust architectural design makes MADet a powerful solution for automated weld inspection. The framework shows significant potential for practical deployment in industrial quality assurance processes, enhancing the efficiency and reliability of non-destructive testing.

In summary, MADet offers a robust and accurate solution for automated weld defect detection, laying a solid foundation for the deployment of advanced AI-powered NDT systems in complex industrial environments.

## 5. Future Work

Despite these promising results, several directions remain for future research and enhancement.

- Edge Deployment: Future work may explore model compression techniques such as quantization and knowledge distillation to further reduce computational cost and enable real-time inference on embedded or edge devices.
- Cross-modality Generalization: While optimized for X-ray imaging, MADet could be extended to other NDT modalities (e.g., ultrasonic or thermal imaging) via domain adaptation or modality-invariant representation learning.
- Fine-grained Classification and Grading: Incorporating defect severity evaluation and conforming to standards such as Technical Specifications for Nondestructive Testing of Pressure Equipment or ASME codes would enhance industrial applicability.Country, Year.
- Few-shot and Self-supervised Learning: To alleviate annotation cost, exploring self-supervised pretraining or few-shot learning methods would benefit rare or underrepresented defect categories.
- Model Interpretability and Uncertainty Estimation: Introducing explainability modules or Bayesian uncertainty frameworks can improve decision confidence and support safety-critical decision-making.

The results suggest that MADet lays a solid foundation for intelligent weld defect detection and opens promising avenues for deploying AI-powered NDT systems in complex industrial environments.

## Figures and Tables

**Table 1:** Classification of weld defects: causes and radiographic characteristics.

Defect Type	Primary Causes	X-Ray Image Characteristics
Crack	High residual stress Hydrogen embrittlement Improper cooling rates	Linear discontinuities with sharp tips Often appears at weld toes or roots
Incomplete Penetration	Insufficient heat input Excessive root gap Incorrect groove angle	Continuous dark line along weld centerline Width proportional to unfused area Distinct edges in butt welds
Lack of Fusion (LOF)	Low welding current Fast travel speed Surface contamination	Irregular planar discontinuities Often parallel to weld bead
Porosity	Gas entrapment Moisture contamination Improper shielding gas	Spherical/oval bright spots Random spatial distribution
Slag Inclusion	Improper slag removal Multi-pass welding defects Incorrect electrode angle	Irregular dark regions Often aligned with weld passes

**Table 2:** Hyperparameter Settings.

Parameter	Value	Description
Image Size	640 × 640 × 3	Input resolution
Learning Rate	0.01	Controls update step size
Batch Size	32	Number of samples per iteration
Epochs	120	Total training cycles
Optimizer	AdamW	Decoupled weight decay optimizer
Random Seed	42	Ensures reproducibility
Data Augmentation	Mosaic	Image augmentation during training
LR Schedule	Step	Step-wise decay for stability

**Table 3:** Dataset Distribution Across Weld Defect Categories.

Item	CK	LOF	LOP	Por	SI	Number of Images
Train	2636	1740	1480	6828	2244	5190
validation	277	183	178	728	238	594
Test	295	182	167	819	245	595

Note: The five typical weld defect categories are denoted as CK (Crack), LOF (Lack of Fusion), LOP (Lack of Penetration), Por (Porosity), and SI (Slag Inclusion).

**Table 4:** Comparison of Detection Performance (mAP@0.5) Across Methods and Defect Categories.

Method	CK	LOF	LOP	Por	SI	mAP@0.5
YOLOv11	77.51	79.58	79.94	84.02	86.04	81.42
Replaced Head	92.59	71.71	82.49	93.91	94.65	87.07
CBAM	92.05	79.86	82.73	94.31	93.81	88.55
CBAM + GAM	92.79	76.89	84.44	**95.32**	**95.89**	89.07
MADet	**94.22**	**80.96**	**90.85**	94.73	94.49	**91.05**

Note: The best results are highlighted in **bold**, while the second-best are underlined.

**Table 5:** Comparison of Detection Performance (mAP@0.5) Across YOLO Versions and Proposed Method.

Method	CK	LOF	LOP	Por	SI	mAP@0.5
YOLOv5	75.44	73.16	67.66	80.09	83.20	75.91
YOLOv6	68.93	56.87	47.07	74.01	78.35	65.04
YOLOv8	80.89	81.13	75.64	84.93	88.09	82.14
YOLOv9	81.91	81.90	78.94	86.59	88.86	83.64
YOLOv10	79.03	79.63	82.79	87.16	78.78	83.43
YOLOv11	77.51	79.58	79.94	84.02	86.04	81.42
YOLOv12	68.98	67.35	67.36	73.77	79.22	71.34
MADet	**94.22**	**80.96**	**90.85**	**94.73**	**94.49**	**91.05**

Note: The best results are highlighted in **bold**, while the second-best are underlined.

## Data Availability

The original contributions presented in this study are included in the article. Further inquiries can be directed to the corresponding author.

## Abbreviations

**Abbreviation**	**Full Name**
AdamW	Adam with Decoupled Weight Decay
AI	Artificial Intelligence
ANN	Artificial Neural Networks
ASME	American Society of Mechanical Engineers
BCE	Binary Cross-Entropy
C2PSA	Cross-Scale Channel Attention
C3K2	A module based on residual connections and cross-channel convolutions
CBAM	Convolutional Block Attention Module
CIoU	Complete Intersection over Union
CK	Crack
CNNs	Convolutional Neural Networks
CPU	Central Processing Unit
CUDA	Compute Unified Device Architecture
DL	Deep Learning
FC	Fully Connected
FPN	Feature Pyramid Network
GAM	Global Attention Mechanism
GPU	Graphics Processing Unit
Grad-CAM	Gradient-weighted Class Activation Mapping
IoU	Intersection over Union
LOF	Lack of Fusion
LOP	Lack of Penetration
LR	Learning Rate
MADet	Multi-Dimensional Feature Fusion Model
mAP@0.5	mean Average Precision (at 0.5 IoU threshold)
MDFFM	Multi-Dimension Feature Fusion Module
MLP	Multi-Layer Perceptron
MT	Magnetic Particle Testing
NB/T	National Board/(Chinese National Standard Prefix)
NDT	Non-Destructive Testing
PAN	Path Aggregation Network
Por	Porosity
PT	Penetrant Testing
R-CNN	Region-based Convolutional Neural Network
ResNet	Residual Network
RT	Radiographic Testing
SAM	Segment Anything Model
SI	Slag Inclusion
SPIE	Society of Photo-Optical Instrumentation Engineers
SPPF	Spatial Pyramid Pooling-Fast
SVM	Support Vector Machines
t-SNE	t-distributed Stochastic Neighbor Embedding
U-Net	(Not an acronym, named for its U-shaped architecture)
UT	Ultrasonic Testing
YOLO	You Only Look Once
